# Shifted termination assay (STA) fragment analysis to detect *BRAF* V600 mutations in papillary thyroid carcinomas

**DOI:** 10.1186/1746-1596-8-121

**Published:** 2013-07-24

**Authors:** So Young Kang, Soomin Ahn, Sun-Mi Lee, Ji Yun Jeong, Ji-Youn Sung, Young Lyun Oh, Kyoung-Mee Kim

**Affiliations:** 1 Department of Pathology, Samsung Medical Center, Sungkyunkwan University School of Medicine, 50 Ilwon-dong, Gangnam-gu, Seoul 135-710, Korea; 2 Department of Pathology, The University of Texas Health Science Center at San Antonio, San Antonio, Texas, USA; 3 Department of Pathology, Kyungpook National University Hospital, Kyungpook National University School of Medicine, Daegu, Korea; 4 Department of Pathology, KyungHee University Medical Center, KyungHee University School of Medicine, Seoul, Korea; 5 Department of Genetic Engineering, Sungkyunkwan University, Suwon 440-746, Korea

**Keywords:** BRAF, Mutation, Termination assay, Sequencing, Thyroid, Papillary carcinoma

## Abstract

**Background:**

*BRAF* mutation is an important diagnostic and prognostic marker in patients with papillary thyroid carcinoma (PTC). To be applicable in clinical laboratories with limited equipment, diverse testing methods are required to detect *BRAF* mutation.

**Methods:**

A shifted termination assay (STA) fragment analysis was used to detect common V600 *BRAF* mutations in 159 PTCs with DNAs extracted from formalin-fixed paraffin-embedded tumor tissue. The results of STA fragment analysis were compared to those of direct sequencing. Serial dilutions of *BRAF* mutant cell line (SNU-790) were used to calculate limit of detection (LOD).

**Results:**

*BRAF* mutations were detected in 119 (74.8%) PTCs by STA fragment analysis. In direct sequencing, *BRAF* mutations were observed in 118 (74.2%) cases. The results of STA fragment analysis had high correlation with those of direct sequencing (p < 0.00001, κ = 0.98). The LOD of STA fragment analysis and direct sequencing was 6% and 12.5%, respectively. In PTCs with pT3/T4 stages, *BRAF* mutation was observed in 83.8% of cases. In pT1/T2 carcinomas, *BRAF* mutation was detected in 65.9% and this difference was statistically significant (p = 0.007). Moreover, *BRAF* mutation was more frequent in PTCs with extrathyroidal invasion than tumors without extrathyroidal invasion (84.7% *versus* 62.2%, p = 0.001). To prepare and run the reactions, direct sequencing required 450 minutes while STA fragment analysis needed 290 minutes.

**Conclusions:**

STA fragment analysis is a simple and sensitive method to detect *BRAF* V600 mutations in formalin-fixed paraffin-embedded clinical samples.

**Virtual Slides:**

The virtual slide(s) for this article can be found here: http://www.diagnosticpathology.diagnomx.eu/vs/5684057089135749

## Introduction

*BRAF* is a serine/threonine kinase that functions as a part of the RAS/RAF/MEK /ERK/MAPK pathway, which is involved in the transduction of mitogenic signals from the cell membrane to the nucleus. A single hotspot mutation at nucleotide 1799 of *BRAF* gene has been identified as the most common genetic event in papillary thyroid carcinoma (PTC) with a prevalence of 29–83% [1]. Recently, systematic review and meta-analyses on PTC showed that *BRAF* mutation is significantly associated with recurrence, lymph node metastasis, extrathyroidal extension and advanced tumor stages [2-4]. So far, detection of this mutation has been achieved by co-amplification at lower denaturation temperature (COLD)-PCR, allele-specific polymerase chain reaction (AS-PCR), high-resolution melting curve (HRM) analysis, SNaPshot Assay, pyrosequencing and direct sequencing. Each technique has advantages and limitations with regard to cost, availability, and enrichment efficiency.

Direct sequencing is widely accepted as the standard for the determination of mutations in formalin-fixed, paraffin-embedded (FFPE) tissue samples although this technique is time consuming, expensive [5,6] and the sensitivity is relatively low [7,8]. A key limitation of PCR-based methods in the detection of *BRAF* mutations is the inability to selectively amplify low percentages of variant alleles from a wild-type allele background [9]. In this study, we first applied shifted termination assay (STA) fragment analysis to detect the common hot-spot *BRAF* mutation in 159 PTCs and compared the results to those of direct sequencing using DNAs from FFPE tissue samples consisting of 53 tumors less than 0.5 cm in size.

## Materials and methods

### Patients and tumor samples

One hundred and fifty nine PTCs were randomly retrieved from the surgical pathology files of Samsung Medical Center, Seoul, Korea between 2010 and 2011. The study was approved by the Institutional Review Board of the Samsung Medical Center (IRB #2009-09-010). The informed consent was obtained from the patient for genetic test and research. A pathologist (Ahn S) performed additional review of each case to confirm diagnosis and select the tumors. The patients included 27 men (17%) and 132 women (83%) with a mean age of 48 years (range, 17–76 years). The mean tumor size was 0.76 cm (range, 0.1-6.5 cm).

### DNA extraction and BRAF mutant cell line

Genomic DNA was extracted from two 4 μm thick sections of FFPE tumor blocks under microscopy as previously described [10] using the QIAamp DNA Mini Kit (QIAGEN, Hilden, Ger.). The concentration and purity of the extracted DNA were determined by a ND-1000 spectrophotometer (NanoDrop Technologies, Inc. Wilmington, DE, USA). The extracted DNA was stocked at 4°C until use.

DNAs from SNU-790 cell lines were used as positive control and normal human genomic DNAs (Roche Applied Science, Penzberg, Ger.) were used as negative control. Serial dilutions of the positive cell line with normal human genomic DNAs to create final tumor DNA concentrations with 100%, 50%, 25%, 12.5%, 5% and 1% were used to compare the analytical limit of detection (LOD).

### Shifted Termination Assay (STA) fragment analysis

Applied Biosystems® *BRAF* mutation analysis reagents assays were used for detection of three different *BRAF* variants (V600E, V600A, and V600G) (Applied Biosystems, CA, USA). The manufacturer’s protocol was followed for the amplification of DNA, clean-up of PCR products, and primer extension reactions. After primer extension, capillary electrophoresis and fragment analysis were performed. PCR reactions were performed in 30 μL volumes using template DNA, *BRAF* PCR primers, and DNA amplification master mix. PCR was performed using a C1000 (Bio-Rad, CA, USA) and PCR cycling conditions were a 5 minute hold at 94°C, followed by 35 cycles of 94°C for 30 seconds, 52°C for 45 seconds, 72°C for 45 seconds, and 72°C for 5 minutes. After PCR, labeled PCR tubes were cleaned up, and 2 μL of the labeled products were mixed with 9.5 μL of HiDi-formamide and 0.5 μL of Genescan SD-130 size standard. The products were separated using a 40 minute run on an ABI Prism 3130 DNA sequencer with POP7 matrix and injection 14 seconds injection time. GeneMapper software, version 4.1 (Applied Biosystems) was used for analysis of the data.

### Polymerase chain reaction (PCR) and direct sequencing

The mutational analyses of *BRAF* exon 15 were performed by direct sequencing of PCR products amplified from genomic DNA, as previously described [10]. PCR was performed in a 20 μL volume containing 100 ng of template DNA, 10х PCR buffer; 0.25 mM dNTPs, 10 pmol primers, and 1.25 U Taq DNA polymerases (iNtRON, Korea). Bi-directional sequencing was performed using the BigDye Terminator v1.1 kit (Applied Biosystems) on an ABI 3130 genetic analyzer (Applied Biosystems). Sequencher version 4.10.1 (Gene Codes Corporation, Ann Arbor, MI, USA) was used along with manual chromatogram reviews. The results were considered mutation-positive if a mutation was detected in both the forward and reverse DNA strands.

### Statistical analysis

Statistical analyses were performed using SPSS statistical software version 19.0 (SPSS, Inc., Chicago, IL, USA). The level of agreement between genotyping findings by different methods was determined with kappa (κ) statistics. Genotyping results were considered concordant in cases of sequence agreement between assays and discordant in cases where no genotype similarity was observed. A p value of <0.05 was considered statistically significant.

## Results

**Figure 1:**
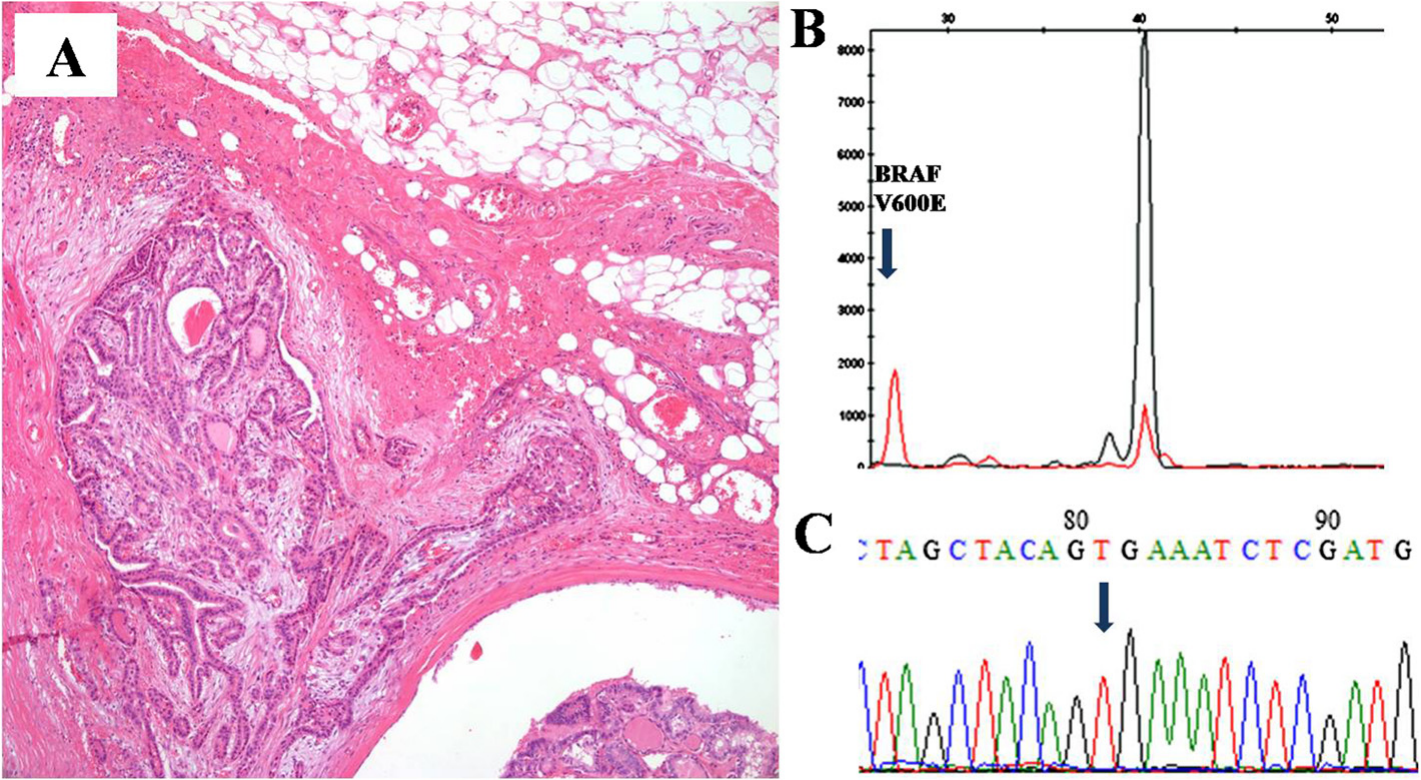
**A papillary thyroid carcinoma with discrepant *BRAF *mutation results in STA fragment analysis and direct sequencing. (A)** Pathology showing extrathyroidal extension. **(B)***BRAF* mutation detected by STA fragment analysis. **(C)** Sequencing result showing wild-type for *BRAF* mutation.

In STA fragment analysis, *BRAF* mutation was detected in 119 (74.8%) PTCs and all mutations were V600E. After serial dilutions of *BRAF* mutant cell line, LOD of STA fragment analysis was 6%. By direct sequencing, *BRAF* V600E mutations were found in 118 (74.2%) cases and LOD was 12.5%. The correlation between STA-fragment analysis and direct sequencing was strong (p < 0.00001) and a high level of agreement was observed [κ, 0.98; 95% CI, (0.98 to 0.99)] (Table 1). Only one case showed discrepant result; a *BRAF* mutation detected by STA fragment analysis was not detected by direct sequencing. Pathologic review of this discrepant case showed extrathyroidal extension and lymph node metastasis in spite of its small size (0.6 cm) (Figure 1).

**Table 1:** **Comparisons of assay performance of direct sequencing *versus *STA fragment analysis and DPO-PCR**

	**Direct sequencing**		**Agreement**
	**V600E**	**Wild type**	
**STAfragment analysis**			
V600E Mutant	118	1	κ, 0.98(95 % CI, 0.98 to 0.99)
wild type	0	40	

The clinicopathological characteristics of patients with *BRAF* V600E mutation detected by direct sequencing are described in Table 2. In PTCs with pT3/T4 stages, *BRAF* mutation was observed in 83.8% of cases. In pT1/T2 carcinomas, *BRAF* mutation was detected in 65.9% and this difference was statistically significant (p = 0.007). Moreover, *BRAF* mutation was more common in PTCs with extrathyroidal invasion than PTCs without extrathyroidal invasion (84.7% *versus* 62.2%, p = 0.001). However, *BRAF* mutation was not significantly associated with patients’ age, tumor size and lymph node metastasis (p > 0.05).

**Table 2:** Clinicopathologic characteristics in 159 patients with papillary thyroid carcinoma

	***BRAF *V600E mutation**		**P value**
**Characteristic**	**Positive**	**Negative**	
Age (y), Mean ± SD	47.70 ± 10.69	48.29 ± 11.62	0.602
Gender			0.422
Male	21	6	
Female	97	35	
Tumor size (cm), Mean ± SD			0.506
<0.5	42	11	
0.5-1	55	20	
>1	21	10	
T classification			**0.007**
T1/T2	56	29	
T3/T4	62	12	
Lymph node metastasis			0.565
Yes	43	15	
No	75	26	
Extrathyroidal extension			**0.001**
Yes	72	13	
No	46	28	

To estimate time and costs for each method, we estimated them from the preparative step to the final interpretation of results. As 3130 ABI sequencer in our laboratory is a 16-channel model, we calculated the sample size at 16. STA fragment analysis required 4 hours and 50 minutes while direct sequencing took 7 hours and 30 minutes. To run 16 samples, STA fragment analysis costs 34$ and direct sequencing requires 25$.

## Discussion

In PTCs, *BRAF* mutation is an important prognostic marker. To detect *BRAF* mutation, direct sequencing has been widely accepted as the gold standard. However, this technique requires rather expensive equipment, can be laborious and time consuming. To be applicable in many clinical laboratories with limited equipments, diverse testing methods are required to detect *BRAF* mutations. For this purpose, we first tested STA fragment analysis to detect *BRAF* V600 mutation in 159 PTCs obtained from FFPE tissue samples and found that STA fragment analysis is as sensitive as direct sequencing and can be easily applicable with lower costs and less running time. Moreover, *BRAF* mutation was associated with extrathyroidal extension and advanced tumor stage in PTCs.

*BRAF* is the strongest activator in the downstream of MAP kinase signaling. In PTCs, the prevalence of *BRAF* mutation has been variable among different studies and its association with clinicopathological features was controversial. Recently, systematic review and meta-analyses on PTC showed that *BRAF* mutation is significantly associated with recurrence, lymph node metastasis, extrathyroidal extension and advanced tumor stages [2-4]. In medullary thyroid carcinoma, RET oncogene mutation correlates with a worse outcome [11]. In this study, although we failed to find the relationship between *BRAF* mutation and lymph node metastasis, we confirmed that *BRAF* mutation is closely associated with extrathyroidal extension and advanced tumor stage. In our previous study on *BRAF* mutations using very highly sensitive dual-priming oligonucleotide-PCR and mutant enrichment with 3′-modified oligonucleotide sequencing in 4,585 consecutive cases in fine needle aspiration cytology specimens, *BRAF* mutation was not significantly associated with pT stage, extrathyroidal extension and lymph node metastasis [12]. In the present study, although the numbers of cases are small, we found that *BRAF* mutation was significantly associated with extrathyroidal extension and advanced tumor stages using the FFPE PTC tissue samples and standard method to detect *BRAF* mutations. These results are consistent with previous observations [2-4].

In order to be applicable in clinical laboratories, the diagnostic assay should address several issues related to the LOD, affordability, turnaround time and running costs. A variety of methods have been applied for *BRAF* mutations. PCR-based screening methods such as SNaPshot assays, AS-PCR, COLD-PCR, Taqman® SNP assay, pyrosequencing and HRM analysis have been applied and the commonly used methods in pathology laboratories are summarized in Table 3[13-18]. Although direct sequencing of PCR products is the gold standard for *BRAF* mutation detection in routine diagnostics, it remains laborious, time consuming and requires rather expensive equipment [5,6]. In this study, we compared LOD, total operation time and costs for the detection of *BRAF* mutation between STA fragment analysis and direct sequencing. STA fragment analysis cannot detect *BRAF* mutations outside the targeted codon although direct sequencing can detect mutations located outside targeted codon. However, in PTCs, *BRAF* V600E is the most common hot spot mutation and was the only mutation found in 159 PTCs in this study. In our study, one PTC with small size (0.6 cm) and wild-type in direct sequencing turned out to harbor *BRAF* V600E mutation by STA fragment analysis. The LOD using a *BRAF* mutant cell line also showed higher sensitivity compared to direct sequencing. In cases with small carcinomas, a more sensitive assay is required to detect rare mutations. In this context, although the numbers of examined cases are small, STA fragment analysis is a sensitive method to detect V600 *BRAF* mutation and needs shorter running time and lower costs compared to direct sequencing. In clinical laboratories performing MSI analyses by fluorescent PCR-based method using an ABI PRISM® 3100, STA fragment analysis can be used as an easily applicable, rapid and cost effective method to detect *BRAF* V600E mutations in FFPE clinical samples.

**Table 3:** **Summary of literature reviews on *BRAF *mutation analyses in recent publications**

**Journal (references)**	**Year**	**Organ**	**Tissue**	**No. of cases**	**Methods**
Present study	2012	Thyroid	FFPE and cell line	159	STA fragment analysis, direct sequencing
J Mod Diagn [19]	2012	Lung and colon	FFPE and cell line	152	HRM-sequencing and HRM-SNaPshot
PLOS One [20]	2011	Brain	FFPE	97	SNaPshot
Eur J Endocrinol [21]	2011	Thyroid	fresh tissues	90	Real-time PCR
PLOS One [8]	2011	Lung	Cytology and FFPE	447 and 42	Real-time PCR
J Mod Diagn [22]	2011	Colon	FFPE	42	Multiplex SNaPshot
Clin Chim Acta [9]	2011	Skin	FFPE	45	COLD-PCR
J Mod Diagn [14]	2011	Colon	FFPE	125	Real-time PCR
Hum Pathol [4]	2010	Thyroid	FFPE	76	RFLP
Genes Chromosomes Cancer	2010	Skin	FFPE	116	conventional PCR

## Abbreviations

DPO-PCR: Dual-priming oligonucleotide-PCR.

## Competing interests

The authors declare that they have no competing interests.

## Authors’ contributions

SYK, KM Kim and YL Oh designed the study and wrote the manuscript. SYK, SA, SML, JYJ, JYS, YL Oh and KMK acquired data. SYK and KMK managed the statistical analysis. All authors read and approved the final manuscript.
